# Collaboration scripts in computer-supported collaborative learning: a systematic review from 2011 to 2024

**DOI:** 10.3389/fpsyg.2026.1717440

**Published:** 2026-07-15

**Authors:** Fangju Jiao, Jiasheng Bian, Rui Zhang, Tong Zhou

**Affiliations:** 1 Graduate School of Education, Hokkaido University, Sapporo, Japan; 2 Preparatory School for Chinese Students to Japan, Northeast Normal University, Changchun, China; 3 Faculty of Education, Northeast Normal University, Changchun, China

**Keywords:** collaboration scripts, computer-supported collaborative learning (CSCL), impacts, learning outcomes, systematic review

## Abstract

Despite its crucial role in fostering learners’ cognitive, social, and affective development, Computer-Supported Collaborative Learning (CSCL) continues to face persistent challenges, such as free-riding and unequal participation, that undermine its effectiveness. Collaboration scripts have been proposed as structured instructional supports that specify roles, sequence interaction, and provide procedural guidance to regulate collaborative processes. However, a comprehensive synthesis of recent empirical evidence remains limited. This study systematically reviews 53 peer-reviewed studies published between 2011 and 2024, examining collaboration scripts across four dimensions: research characteristics, learning outcomes, measurement approaches, and influencing factors. The findings indicate fluctuating publication trends, with renewed interest since 2020 driven by adaptive scripting and generative AI-supported approaches, reflecting a shift toward more flexible and learner-centered designs. Overall, collaboration scripts are associated with improvements across cognitive, behavioral, affective, and skills domains, though evidence regarding affective and skills-related outcomes remains mixed. Methodologically, studies rely on diverse instruments—including tests, questionnaires, user log data, artefacts, and interviews—yet single-method assessments are still common. Synthesizing influencing factors, this review conceptualizes collaboration scripts as regulation-distribution mechanisms whose impact emerges from the alignment among external supportive structures, learners’ internal regulatory capacities, and contextual conditions. These findings provide theoretical and practical implications for the design and implementation of collaboration scripts in CSCL and suggest directions for future research.

**Systematic Review registration:**

10.17605/OSF.IO/VQFYC; https://osf.io/vqfyc/

## 1 Introduction

Computer-supported collaborative learning (CSCL) investigates the use of digital tools in collaborative learning and has been widely recognized for its potential to enhance cognitive, social, and affective outcomes (Chen et al., 2018; Miller and Hadwin, 2024; Reis et al., 2018). From a cognitive perspective, CSCL promotes the acquisition of specific knowledge and maximizes learning achievement (Chen et al., 2021). Socially, it facilitates interaction and co-regulation among learners (Dado and Bodemer, 2017; Miller and Hadwin, 2024). In addition, affective outcomes such as motivation and self-efficacy have been linked to well-designed CSCL environments (Chen and Yeh, 2024; Jeong et al., 2019).

However, effective collaboration in CSCL does not emerge spontaneously (Chen et al., 2021; Kollar et al., 2007; Nussbaum et al., 2009). Without deliberate structuring, learners may experience challenges such as unequal participation (Caspi et al., 2003), free-riding (Hämäläinen and Arvaja, 2009), or the presence of lurkers and silent participants (Persico et al., 2010), even in technology-rich environments.

To address these challenges, collaboration scripts have been proposed as structured instructional supports designed to scaffold learners’ interactions in CSCL settings (Fischer et al., 2013; Kollar et al., 2006). Typically, collaboration scripts specify roles, sequence activities, and provide procedural guidance to regulate discourse and coordinated action (Kollar et al., 2007). By structuring how learners interact and engage with tasks, collaboration scripts aim to foster deeper knowledge construction and more effective collaboration processes (Dillenbourg and Fischer, 2007).

A growing body of empirical research has examined the effectiveness of collaboration scripts. Meta-analyses and systematic reviews have reported small-to-moderate positive effects on domain-specific knowledge, collaborative skills, and motivational outcomes (Radkowitsch et al., 2020; Vogel et al., 2017). More recently, Cai et al. (2025) reviewed adaptive collaboration scripts, identifying specific conditions under which fading or adaptable scripts may be beneficial.

Nevertheless, several gaps remain. First, most prior syntheses have primarily focused on effect size aggregation, providing limited descriptive analysis of study characteristics, outcome domains, and measurements. Second, many reviews synthesized studies published before 2020, leaving more recent developments insufficiently integrated. Third, the most recent review focused exclusively on adaptive scripts and was based on a relatively small sample (11 studies), limiting the generalizability of its conclusions across broader script types and implementation contexts. Importantly, although individual studies have highlighted contextual and design-related factors affecting the impacts of script, these factors have not been systematically synthesized across recent research. Consequently, the field lacks an integrated and comprehensive understanding of how study characteristics, learning outcomes, measurements, and factors collectively shape reported findings.

To address these limitations, the present study conducts a systematic review of research on collaboration scripts in CSCL published between 2011 and 2024. Given the diversity in research designs, learning contexts, outcome domains, and assessment approaches, an exclusive focus on effect-size aggregation would provide only a partial representation of the field. Instead, a systematic review with qualitative synthesis enables a structured mapping of how collaboration scripts are studied, what learning outcomes are reported, how these outcomes are measured, and what factors are identified across studies.

Accordingly, this review aims to identify trends in study characteristics, categorize reported learning outcomes, summarize measurement approaches, and synthesize reported influencing factors, using a hybrid content analysis approach that combines deductive and inductive procedures.

Typically, the study seeks to answer the following research questions:

RQ1: What are the characteristics of studies included in this review?

RQ2: What types of learning outcomes are associated with collaboration scripts in CSCL?

RQ3: How are these learning outcomes measured?

RQ4: What factors influence the impacts of collaboration scripts on learning outcomes in CSCL?

## 2 Methods

**Figure 1:**
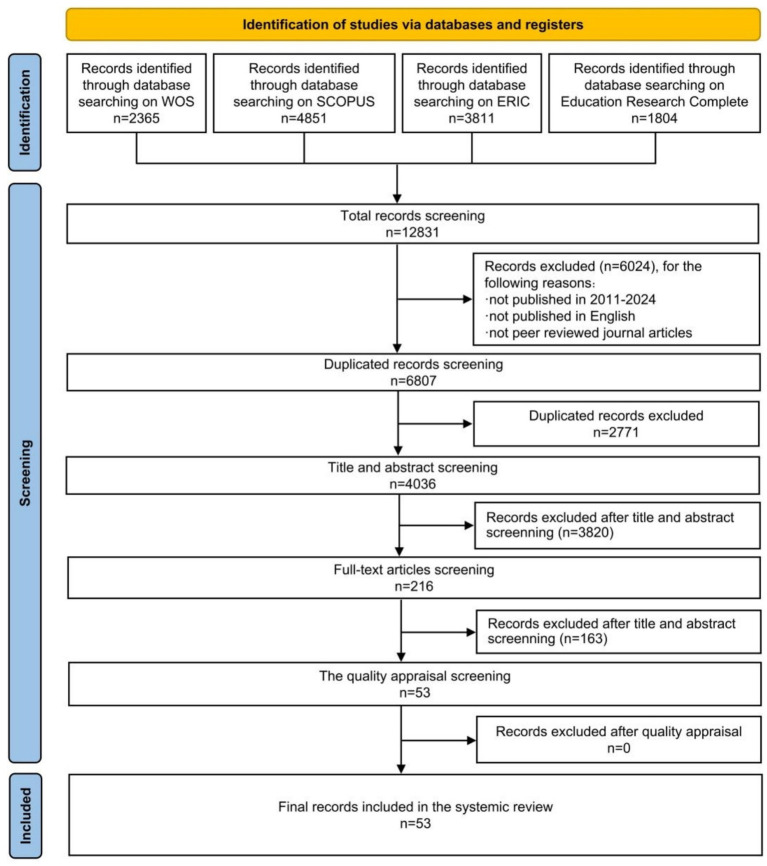
PRISMA flow diagram of the selection process.

Given that the four research questions focus on synthesizing evidence across diverse empirical studies, a systematic review following the Preferred Reporting Items for Systematic Reviews and Meta-Analyses (PRISMA) guidelines and checklist (Page et al., 2021) was considered the most appropriate methodology. This approach allows for comprehensive and transparent integration of heterogeneous findings and ensures methodological rigor through predefined search procedures, eligibility criteria, and independent screening. The literature screening process was carried out independently by two researchers with extensive experience in CSCL research. Disagreements were discussed and resolved through consensus. Figure 1 demonstrates the detailed procedures for this systematic review and the number of papers from initial identification to the final inclusion.

### 2.1 Search strategy

Considering the comprehensiveness and relevance, this systematic review chose four databases as the information sources, which include Web of Science (WOS), Scopus, Educational Resources Information Center (ERIC) and Education Research Complete (ERC). The WOS and the Scopus are the most widespread and the largest databases on different scientific fields (Chadegani et al., 2013; Chang et al., 2022), while the ERIC and the ERC are major scientific databases in educational research (Courtney et al., 2023).

To identify relevant studies, we conducted comprehensive searches using Boolean operators and truncation symbols (*) across selected databases. The search strategy was developed based on established reviews and meta-analyses on collaboration scripts (Radkowitsch et al., 2020; Vogel et al., 2017) and CSCL (Chen et al., 2018; Jeong et al., 2019), and was structured around two conceptual domains (see Table 1): Script-related terms and CSCL-related terms. The final Boolean structure applied in each database was: (Script-related terms) AND (CSCL-related terms). Field specifications (e.g., title, abstract, and keywords) were adjusted according to the syntax requirements of each database to ensure transparency and replicability. The search was conducted on 1 September 2024 and a total of 12,831 studies were identified.

**Table 1:** Search strings included in this systematic review.

Conceptual domains	Search strings
Script-related terms	(scaffold* OR script*)
CSCL-related terms	CSCL* OR “Computer Supported Collaborative Learning” OR “Computer Supported Cooperative Learning” OR ( (ICT* OR computer* OR techno* OR web* OR internet* OR network* OR mobile* OR virtual* OR simulat* OR game* OR text*) AND (collaborat* OR cooperat* OR synerg* OR coordina* OR group* OR team*) AND learn* )

### 2.2 Eligibility criteria

In order to ensure the relevance and validity, we adopted nine criteria to select articles. The inclusion criteria and exclusion criteria have been demonstrated in Table 2. The entire process of screen could be divided into four stages. Firstly, three filter conditions were set in the four databases: (1) the time interval was limited from 1 January 2011 to 31 December 2024. The starting year was selected to capture developments in collaboration script research over the past decade and to build upon earlier meta-analytical work (Radkowitsch et al., 2020; Vogel et al., 2017), while the end year ensured inclusion of the most recent studies, including emerging approaches such as AI-supported scripting. (2) Papers were published in English. (3) The type of the papers were peer-reviewed journal articles rather than conference paper, editorial, book chapters and so on. Out of the 12,831 records initially retrieved, 6,807 articles remained to the next step of screening while 6,024 records were excluded. Secondly, we imported the above remained records to the Zotero software and Covidence online application, respectively, for removing duplicates. As a result, a total 4,036 articles were eligible for the titles and abstracts review after duplicate removal. Thirdly, based on nine eligibility criteria, the first two authors screened 20% of the 4,036 selected titles and abstracts using the Covidence online application and achieved an inter-rater reliability of Cohen’s kappa = 0.820. Any disagreement in the review of titles and abstracts was resolved through discussion between two researches, resulting in a consensus. Then, the first author checked the remaining 80% of the 4,036 chosen articles to determine the included articles for the full-text screening. This step resulted in 3820 studies being excluded, and 216 articles would be assessed in the next step. Lastly, the two researchers read the 216 full-text articles based on the inclusion and exclusion criteria. The Cohen’s kappa was calculated to ensure the inter-rater reliability with result of 0.826, representing a high level of agreement (McHugh, 2012). After discussing and solving the conflicts, a total of 53 papers were finally included in this systematic review.

**Table 2:** Inclusion criteria and exclusion criteria in this systematic review.

Inclusion	Exclusion
Published between 2011 and 2024	Published before 1 January 2011 or after 31 December 2024
Written in English	Written in languages other than English
Indexed by SCI or SSCI	Not indexed by SCI or SSCI, for example papers were indexed by ESCI
Available for full text	Not available for full text
Focused on the collaboration scripts	Not focused on the collaboration scripts
Focused on the CSCL	Not focused on the CSCL
Reported the effects of collaboration scripts in CSCL	Not reported the effects of collaboration scripts in CSCL

### 2.3 Quality appraisal

The methodological quality of the 53 included studies was assessed using the Mixed Methods Appraisal Tool (Hong et al., 2018), a critical appraisal instrument specifically developed for systematic reviews incorporating qualitative, quantitative, and mixed methods research.

The MMAT comprises two stages. First, all empirical studies are evaluated using two screening questions: (1) whether the study has clear research questions, (2) whether the collected data adequately address those research questions. Only studies meeting both screening criteria proceed to the second stage. In the second stage, studies are categorized according to their methodological design and assessed using one of five sets of design-specific criteria: (a) qualitative research, (b) randomized controlled trials, (c) non-randomized studies, (d) quantitative descriptive studies, (e) mixed methods studies. For mixed methods studies, the five mixed methods criteria are applied in addition to the relevant qualitative and quantitative components, following MMAT guidelines. Each criterion is rated as “Yes,” “No,” or “Cannot tell.”

Two researchers independently conducted the full MMAT appraisal for all 53 studies. Inter-rater reliability was calculated using Cohen’s kappa (*K* = 0.835), indicating substantial agreement. Discrepancies were discussed in structured consensus meetings and resolved through mutual agreement before final ratings were confirmed.

Overall, the methodological quality was moderate to high across study designs. All qualitative studies (*n* = 3) met the appraisal criteria. Randomized controlled trials (*n* = 28) generally reported appropriate randomization and intervention adherence, although limitations were observed in outcome completeness and blinding. Non-randomized studies (*n* = 18) demonstrated adequate measurement and control of confounders, while participant representativeness and completeness of outcome data were less consistently addressed. Mixed-methods studies (*n* = 4) largely met integration and methodological criteria. Detailed appraisal results are presented in Table 3.

**Table 3:** Quality appraisal results of included studies.

Category of study designs	Number		Methodological quality criteria	Yes (*n*)	Yes (%)
Qualitative	3		1.1. Is the qualitative approach appropriate to answer the research question?	3	100.00%
			1.2. Are the qualitative data collection methods adequate to address the research question?	3	100.00%
			1.3. Are the findings adequately derived from the data?	3	100.00%
			1.4. Is the interpretation of results sufficiently substantiated by data?	3	100.00%
			1.5. Is there coherence between qualitative data sources, collection, analysis and interpretation?	3	100.00%
Quantitative randomized controlled trials	28		2.1. Is randomization appropriately performed?	28	100.00%
			2.2. Are the groups comparable at baseline?	25	89.29%
			2.3. Are there complete outcome data?	13	46.43%
			2.4. Are outcome assessors blinded to the intervention provided?	6	21.43%
			2.5 Did the participants adhere to the assigned intervention?	28	100.00%
Quantitative non randomized	18		3.1. Are the participants representative of the target population?	9	50.00%
			3.2. Are measurements appropriate regarding both the outcome and intervention (or exposure)?	18	100.00%
			3.3. Are there complete outcome data?	8	44.44%
			3.4. Are the confounders accounted for in the design and analysis?	18	100.00%
			3.5. During the study period, is the intervention administered (or exposure occurred) as intended?	18	100.00%
Mixed methods	4	3 (Qualitative + Quantitative non randomized)	1.1. Is the qualitative approach appropriate to answer the research question?	3	100.00%
			1.2. Are the qualitative data collection methods adequate to address the research question?	3	100.00%
			1.3. Are the findings adequately derived from the data?	3	100.00%
			1.4. Is the interpretation of results sufficiently substantiated by data?	2	66.67%
			1.5. Is there coherence between qualitative data sources, collection, analysis and interpretation?	3	100.00%
			3.1. Are the participants representative of the target population?	1	33.33%
			3.2. Are measurements appropriate regarding both the outcome and intervention (or exposure)?	3	100.00%
			3.3. Are there complete outcome data?	2	66.67%
			3.4. Are the confounders accounted for in the design and analysis?	1	33.33%
			3.5. During the study period, is the intervention administered (or exposure occurred) as intended?	2	66.67%
			5.1. Is there an adequate rationale for using a mixed methods design to address the research question?	3	100.00%
			5.2. Are the different components of the study effectively integrated to answer the research question?	3	100.00%
			5.3. Are the outputs of the integration of qualitative and quantitative components adequately interpreted?	2	66.67%
			5.4. Are divergences and inconsistencies between quantitative and qualitative results adequately addressed?	3	100.00%
			5.5. Do the different components of the study adhere to the quality criteria of each tradition of the methods involved?	3	100.00%
		1 (Qualitative + Quantitative descriptive)	1.1. Is the qualitative approach appropriate to answer the research question?	1	100.00%
			1.2. Are the qualitative data collection methods adequate to address the research question?	1	100.00%
			1.3. Are the findings adequately derived from the data?	1	100.00%
			1.4. Is the interpretation of results sufficiently substantiated by data?	1	100.00%
			1.5. Is there coherence between qualitative data sources, collection, analysis and interpretation?	1	100.00%
			4.1. Is the sampling strategy relevant to address the research question?	1	100.00%
			4.2. Is the sample representative of the target population?	0	0.00%
			4.3. Are the measurements appropriate?	1	100.00%
			4.4. Is the risk of nonresponse bias low?	1	100.00%
			4.5. Is the statistical analysis appropriate to answer the research question?	1	100.00%
			5.1. Is there an adequate rationale for using a mixed methods design to address the research question?	1	100.00%
			5.2. Are the different components of the study effectively integrated to answer the research question?	1	100.00%
			5.3. Are the outputs of the integration of qualitative and quantitative components adequately interpreted?	1	100.00%
			5.4. Are divergences and inconsistencies between quantitative and qualitative results adequately addressed?	1	100.00%
			5.5. Do the different components of the study adhere to the quality criteria of each tradition of the methods involved?	1	100.00%

### 2.4 Data coding and analysis

To address the research questions, a structured coding scheme was developed to systematically extract and analyze data from the included studies. The coding scheme consisted of four categories.

1. Characteristics of selected studies. This category focused exclusively on publication and research design features, including year of publication, geographical region, research method, sample size, group size and the education level of participants.
2. Learning outcomes. Based on existing theoretical frameworks of learning outcomes (Kraiger et al., 1993; Post et al., 2019), learning outcomes were deductively classified into four domains: cognitive outcomes (e.g., knowledge acquisition, cognitive strategies), skill-based outcomes (e.g., collaborative skills; regulation skills), behavioral outcomes (e.g., engagement, interactions), and affective outcomes (e.g., attitudes, motivation). The impacts of collaboration scripts were further categorized as positive, negative, mixed, or non-significant.
3. Measurements of learning outcomes. This category documented the instruments and data sources used to assess learning outcomes, including quantitative measures (e.g., standardized tests, questionnaires) and qualitative approaches (e.g., coded artifacts, interviews).
4. Factors influencing the impacts of collaboration scripts on learning outcomes. Unlike the previous categories, this part of the analysis adopted an inductive content analysis approach to identify factors reported to influence the effects of collaboration scripts in CSCL.

Overall, a hybrid content analysis approach was adopted, combining deductive (directed content analysis) and inductive (conventional content analysis) procedures (Hsieh and Shannon, 2005). Deductive coding was applied to three predefined categories—Characteristics of selected studies, Learning outcomes, and Measurements of learning outcomes—which were developed based on existing theoretical frameworks and prior literature. In contrast, the category of Factors influencing the effects of collaboration scripts was analyzed inductively. Following the procedures described by Elo and Kyngas (2008), open coding was conducted to identify meaningful units related to influencing factors without imposing any predefined theoretical structure, which were then grouped into sub-categories and abstracted into higher-level categories.

The analysis proceeded in several steps. First, two researchers independently read all included studies to gain familiarity with the data. Second, relevant information was extracted according to the predefined coding scheme. For the inductive component (influencing factors), qualitative content analysis was conducted using NVivo 14 to facilitate data organization and coding. At this stage, meaningful units related to factors influencing the reported effects were identified and coded through open coding. Third, similar codes were grouped into subcategories based on conceptual similarity. Finally, through abstraction, higher-level categories were constructed by examining relationships among subcategories and identifying overarching dimensions that captured common characteristics. The decision to retain a four-category structure was based on conceptual coherence, distinctiveness between categories, and saturation of themes.

The two researchers compared their coding results at each stage, and discrepancies were resolved through iterative discussion until consensus was reached to enhance the reliability and credibility of the analysis.

## 3 Results

This section presents the findings of a systematic review of 53 selected publications on collaboration scripts in CSCL, organized according to the four research questions guiding this study. Section 3.1 provides a descriptive overview of study characteristics (RQ1), offering contextual information about publication and research design features. Sections 3.2 and 3.3 present analytic syntheses of learning outcomes (RQ2) and their measurements (RQ3), respectively. Section 3.4 addresses RQ4 and presents an inductive synthesis of factors influencing the impact of collaboration scripts on learning outcomes.

### 3.1 Characteristics of selected studies

#### 3.1.1 Publication information

**Figure 2:**
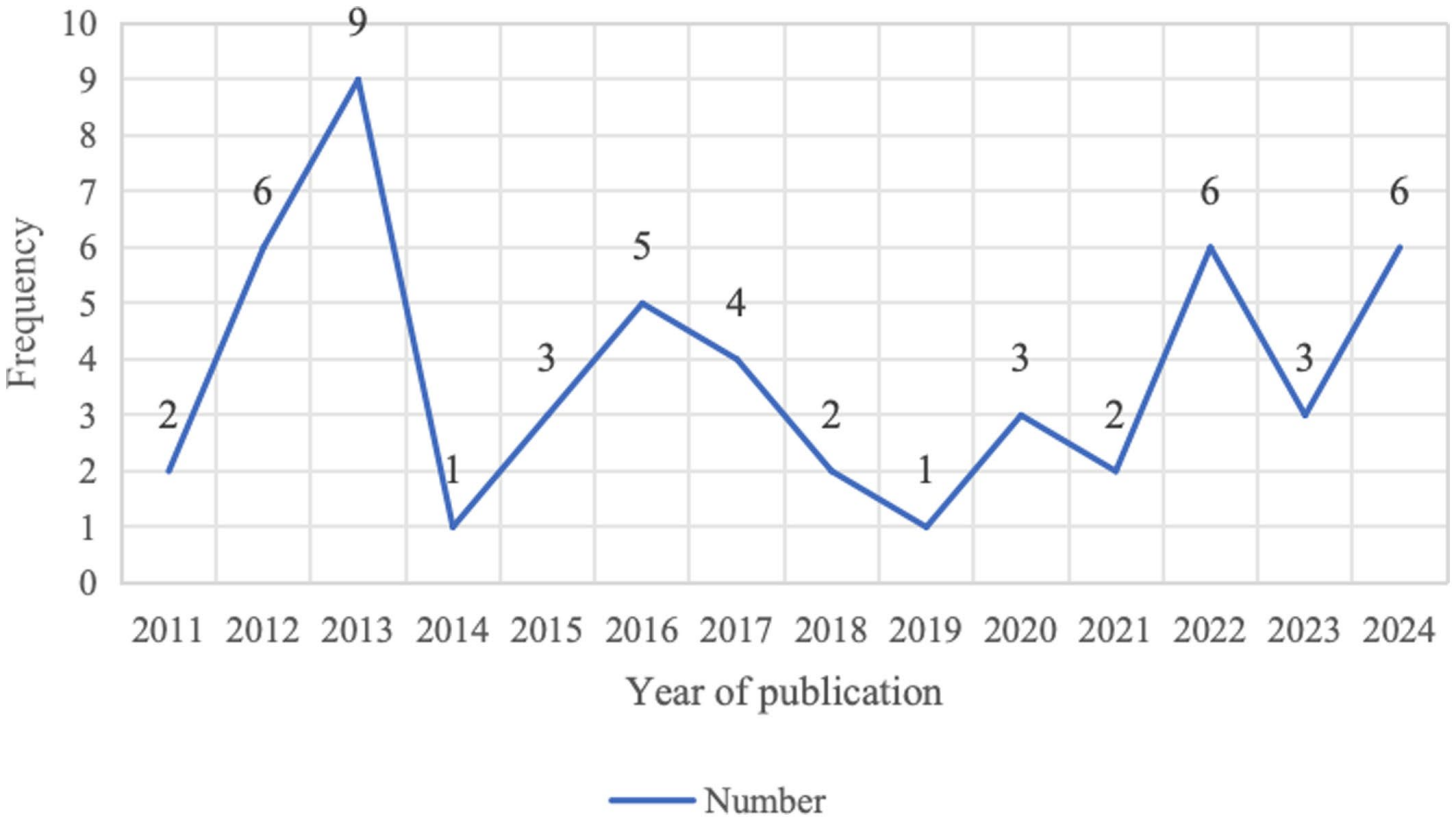
Distribution of articles included in this review by year of publication (2011–2024).

The findings show that the year of publications on collaboration scripts in CSCL fluctuates from 2011 to 2024 (see Figure 2). There was a significant increase in articles between 2011 and 2013, with a peak of 9 articles in 2013, while there was a sharp decrease in the number of studies between 2013 and 2014, with the lowest number (n = 1) in 2014. Furthermore, after reaching the lowest point in 2014, the number of publications generally recovered over the following years and exhibited a fluctuating upward trend between 2020 and 2024.

**Figure 3:**
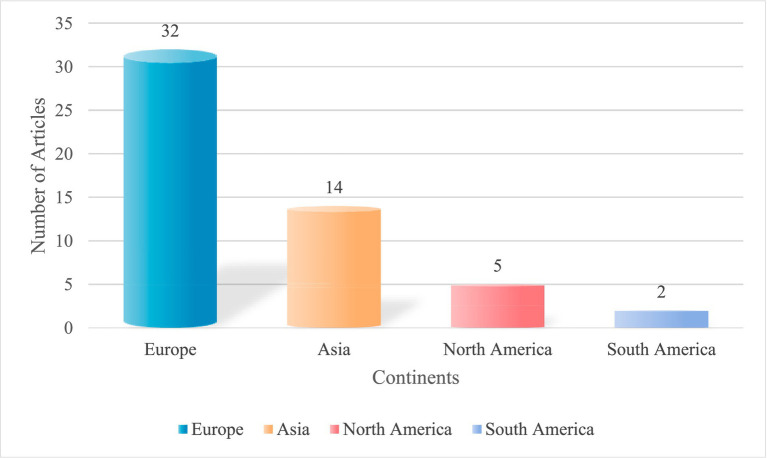
Distribution of articles on collaboration scripts in CSCL by continents (2011–2024).

As shown in Figure 3, the included articles comprised research primarily from four continents (based on the first author’s affiliation): Europe (*n* = 32), Asia (*n* = 14), North America (*n* = 5), and South America (*n* = 2). It is evident that research into collaboration scripts is predominantly conducted in Europe. Of these, the most studies from the Netherlands and Germany with 11 publications accounting for approximately 21% of total, respectively. However, collaboration scripts in the global south (such as Africa), has not yet been adequately investigated.

#### 3.1.2 Research design-related information

**Figure 4:**
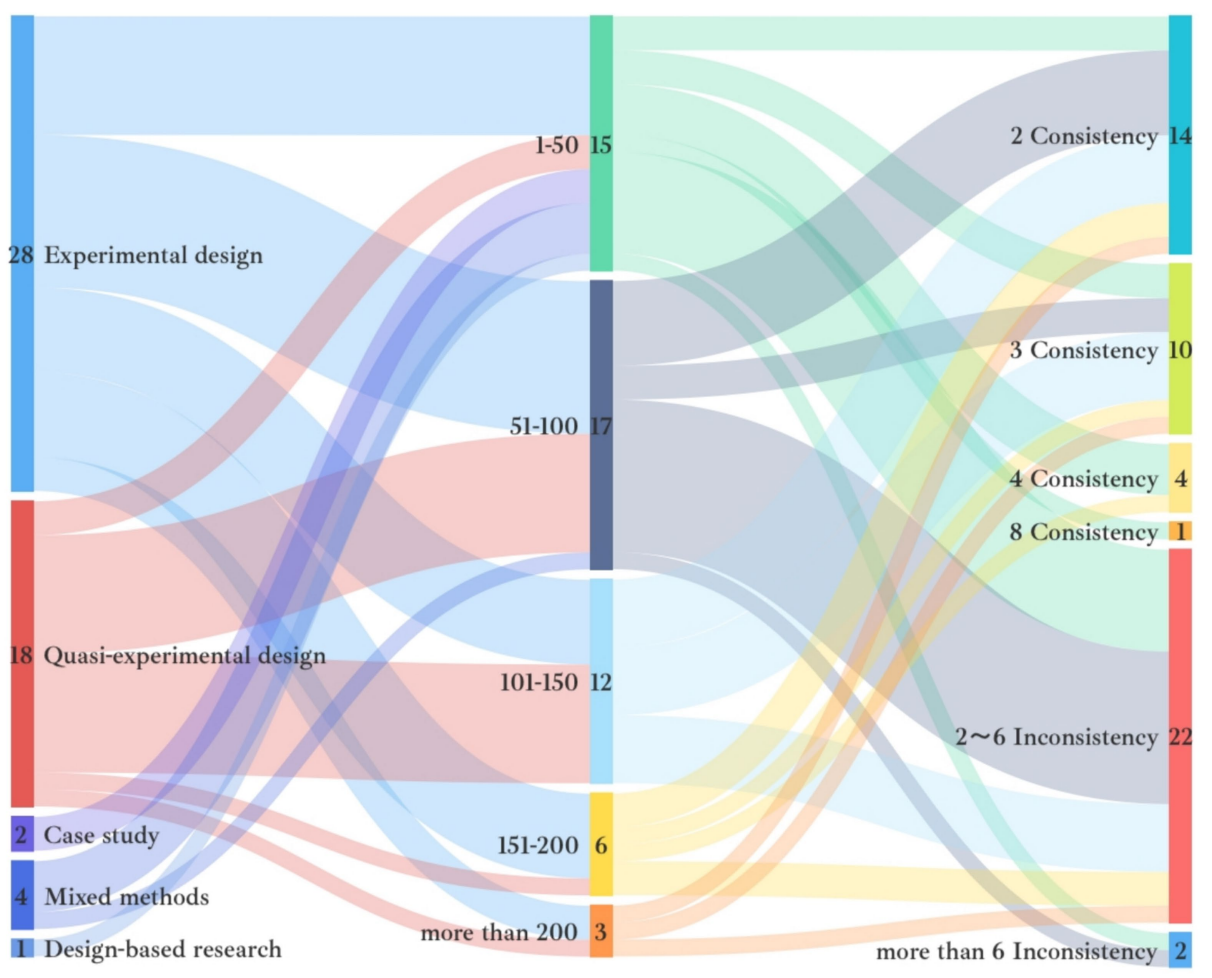
Distribution of articles on collaboration scripts in CSCL by types of methods, sample size, and group size (2011–2024).

As shown in Figure 4, the majority of the 53 reviewed studies adopted quantitative methodologies, with 52.8% (*n* = 28) employing experimental designs involving random assignment to groups and conditions, followed by 34% (*n* = 18) utilizing quasi-experimental approaches. In these studies, some employed one-factor experimental designs, which compared groups under the guidance of collaboration scripts with control groups without them (Chen and Chiu, 2016; Lee, 2015), while others adopted multi-factorial designs to examine the roles of collaboration scripts and additional elements (Peterson and Roseth, 2016; Weinberger et al., 2013). A limited number of studies employed mixed methods (*n* = 4), combining quantitative analysis with qualitative insights to deepen understanding. In addition, three studies employed qualitative designs, comprising two case studies and one design-based research study.

The sample size of included studies was wide-ranging, from 8 to 617 participants. As shown in Figure 4, most of research involved the number of participants between 51 and 100 (*n* = 17). A total of 15 publications were based on relatively small numbers of participants (1–50 participants). Twelve and 6 articles examined collaboration scripts with 101–150 and 151–200 participants, respectively. Only three studies included more than 200 participants. In addition, studies with group sizes ranging 2–6 were the most (*n* = 22). Group sizes of two (*n* = 14) and three (*n* = 10) constituted the second and third most prevalent categories, respectively.

**Figure 5:**
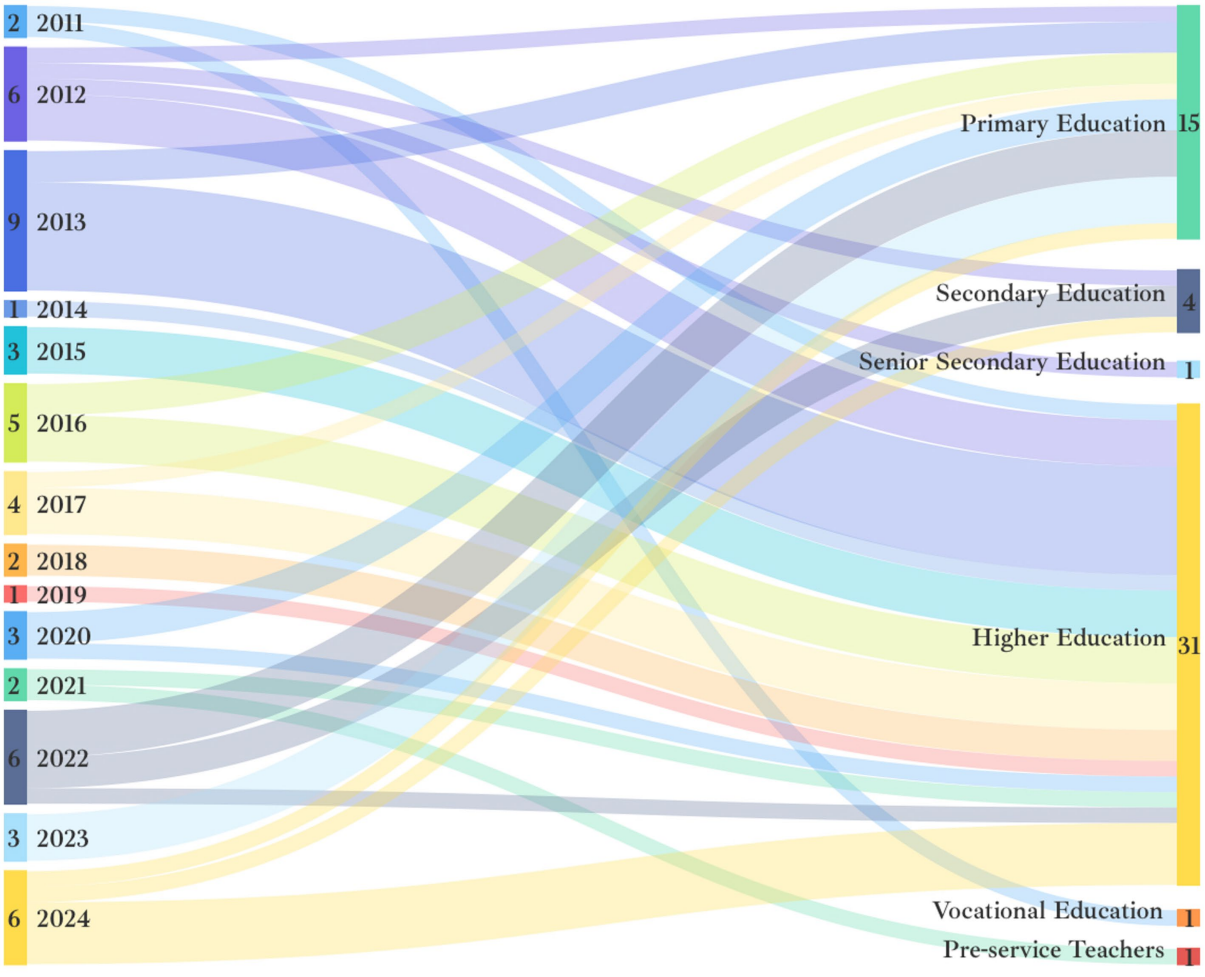
Percent of articles on collaboration scripts in CSCL by educational levels of participants (2011–2024).

From the educational levels of participants (Figure 5), 52 publications focused on the students from primary schools to universities, while only one study concentrated on the pre-service teachers with bachelor’s or master’s degrees. Of the 53 articles included in this study, 31 (approximately 58%) were mainly conducted in higher education, ranging from undergraduate to doctoral levels. In addition, 15 and 4 publications, respectively, examined the collaboration scripts in primary school and secondary school, accounting for 28 and 8% of the total for each. Only 1 for each of the studies focused on collaboration scripts for high schools and vocational schools. Furthermore, K–12 educational settings have received increasing research attention in recent years, particularly after 2020.

### 3.2 Learning outcomes of collaboration scripts in CSCL

**Figure 6:**
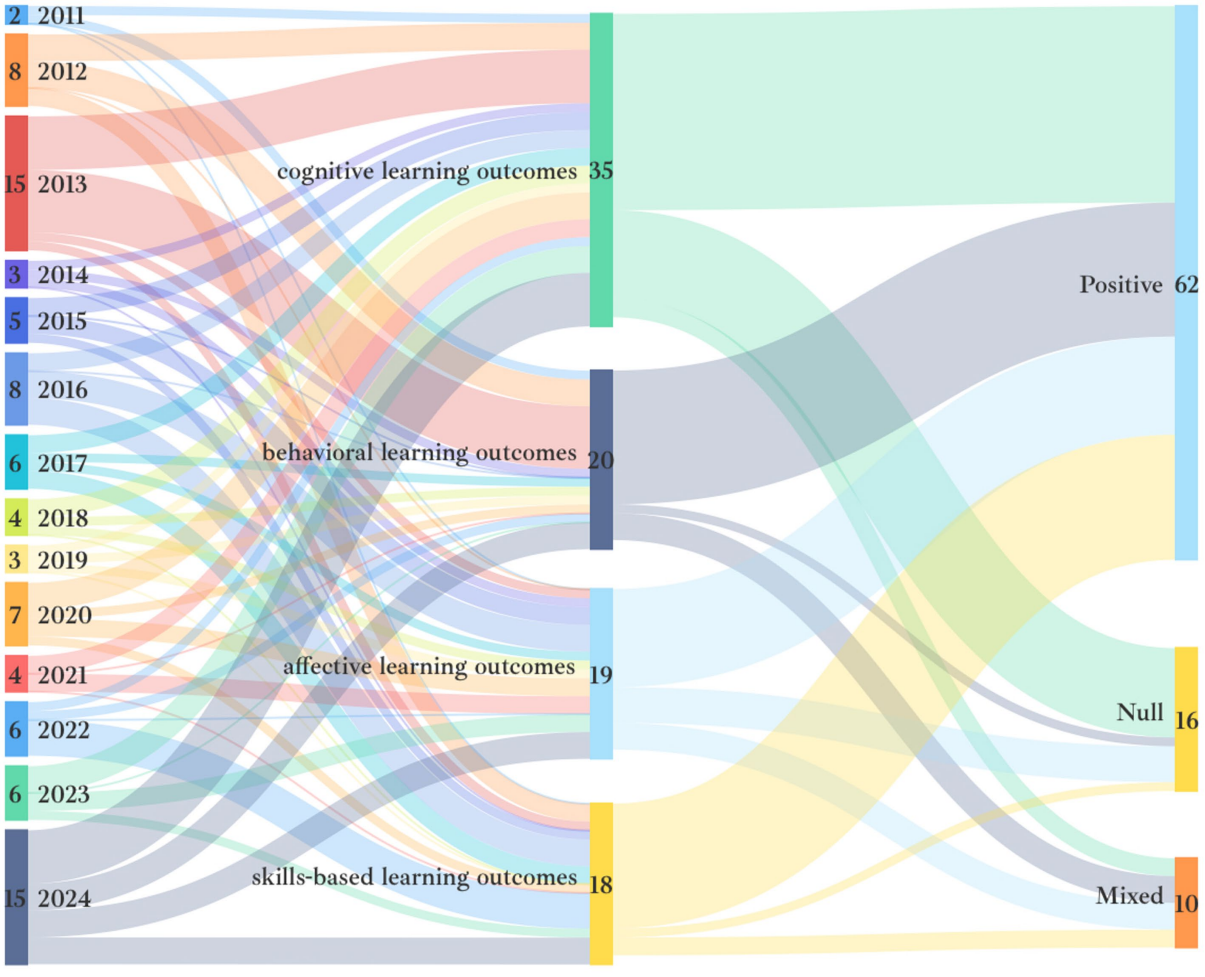
Relationships among publication year, learning outcome category, and the reported effects of collaboration scripts in CSCL (2011–2024).The flow values in this Sankey diagram represent the total number of learning outcome measurement instances rather than the number of included studies. Because a single study may assess multiple learning outcome categories, it may contribute to multiple flows throughout the diagram.

To address RQ2, the learning outcomes reported in the selected studies were identified and categorized into four types (Figure 6): cognitive outcomes, behavioral outcomes, affective outcomes, and skills-based outcomes. Cognitive outcomes (*n* = 35) were the most frequently examined, whereas skills-based outcomes (*n* = 18) were the least reported.

Cognitive outcomes. Cognitive outcomes refer to improvements in understanding and knowledge associated with cognitive development (Rogaten et al., 2018). In 35 studies (see Table 4), cognitive learning outcomes were measured primarily in terms of knowledge acquisition, learning performance, and academic achievement. The sub-category of knowledge referred to the acquisition of specific-knowledge, such as the argumentation knowledge (Stegmann et al., 2012; Vogel et al., 2021), conception knowledge of photosynthesis (Gijlers et al., 2013), the acquisition of math (Chen and Chiu, 2016), and vocabulary (Chen and Yeh, 2024). The reported effects of collaboration scripts on these domains were mixed: 22 studies reported positive impacts (e.g., Slof et al., 2012), while 11 studies found non - significant effects (e.g., Cáceres et al., 2018; Dlab et al., 2020; Popov et al., 2019).

**Table 4:** An overview of cognitive learning outcomes in the studies reviewed.

Authors (year)	Type of outcome	Instruments	Findings
Bouyias and Demetriadis (2012)	Knowledge	Tests	Null: the fading of the micro-script does not have a significant impact on domain leaning.
Chen and Chiu (2023)	Knowledge	Tests	Positive: have better learning achievement.
Cáceres et al. (2018)	Learning performance	Tests	Positive: Collpad 2 + Arg group ≥ Control group (without technology).
Chen et al. (2021)	Knowledge	Artifacts (design)	Positive: the quality of TEL design at each phase was always higher than those of the preceding phases.
Chen et al. (2023)	Knowledge	Artifacts	Positive: students’ knowledge of the subject studies did improve in this study.
Chen and Yeh (2024)	Knowledge	Tests	Positive: The post-test score of the scripted collaborative DST group was statistically higher than that of the collaborative DST group, indicating a large effect size.
Darmawansah et al. (2024)	Argumentative speaking performance	Tests	Positive: higher mean scores.
De Weyer et al. (2015)	Knowledge	Tests	Null: although the increase was higher in the scripted condition but the difference between the conditions was not statistically significant.
Gijlers et al. (2013)	Knowledge	Artifacts (drawing quality); tests	Positive: the scripted students acquired significantly more conceptual knowledge than the unscripted students.
Heimbuch and Bodemer (2018)	Knowledge	Test	Null effects: the explicit scripting group equal to the implicit controversy highlight group
Heimbuch et al. (2018)	Learning gains	Tests	Null: performed minimally better, but the magnitude of the scripting effect was negligibly small.
Dlab et al. (2020)	Knowledge	User data (log files; lesson video recordings)	Mixed: for tasks of lower difficulty, task distribution using roles led to statistically significantly more incorrect task completion attempts, for tasks of greater difficulty, there were more incorrect task completion attempts in groups with no explicit distribution.
Hummel et al. (2015)	Learning performance	Artifacts (reports)	Positive: most participants’ reports could be graded as sufficient.
Hung et al. (2024)	Knowledge	Tests	Positive: knowledge improved significantly.
Kopp and Mandl (2011)	Knowledge	Artifact (task solutions); tests	Positive: positive effects of collaboration scripts on argument justification.
Lin (2020)	Learning performance	Artifacts (report)	Null: no significant differences between the grades of students.
Marée et al. (2013)	Knowledge	Exams; tests	Positive: more effective.
Van der Meij et al. (2020)	Knowledge	Tests	Positive: players in the scripted condition achieved a significantly higher mean score on a knowledge posttest.
Miller and Hadwin (2024)	Group performance	Artifacts (assignments)	Null: did not appear to influence group performance.
Noroozi et al. (2013)	Knowledge	Tests	Positive: the transactive discussion script fostered the acquisition of knowledge on single arguments, the individual knowledge acquisition on argumentation sequences and individual acquisition of domain-specific knowledge.
Peterson and Roseth (2016)	Learning achievement	Exams	Positive: higher academic achievement.
Popov et al. (2014)	Learning performance	Artifacts (project plans)	Positive: higher scores.
Popov et al. (2019)	Learning performance	Artifacts (assignments)	Null: no significant differences were found between the IECS and CS conditions.
Rau et al. (2017)	Knowledge	Tests	Positive: significantly higher learning gains.
Slof et al. (2012)	Learning performance	Assessments	Positive: performed better.
Stegmann et al. (2012)	Knowledge; cognitive elaboration	Tests; user data	Positive: positive: with respect to knowledge on argumentation-with scripts ≥ without scripts; with respect to the depth of cognitive elaboration-with scripts ≥ without scripts; positive but: with respect to domain-specific knowledge-with scripts ≤ without scripts.
Scheuer et al. (2013)	Knowledge	Tests	Null: no difference in terms of detailed knowledge of the given texts.
Strauß et al. (2024)	Knowledge	Tests	Null: external collaboration script did not lead to a larger increase in knowledge
Tsompanoudi et al. (2016)	Learning performance	Pass rates; final exams	Positive: pass rate in 2014 is significantly higher than in previous years; had a significant better overall performance.
Vogel et al. (2021)	Knowledge	Tests; final exams	Positive: argumentation knowledge can be supported with CSCL scripts that are designed at a dedicated level.
Wang et al. (2023)	Knowledge; cognitive load	Tests	Mixed: null: external scripts availability had no significant effect on knowledge acquisition; mixed: no significant effect on the mental load, but a higher effect on mental effort.
Wang et al. (2023)	Knowledge	Tests	Null: no significant difference in students’ inquiry performance and knowledge acquisition under different settings with or without external scripts.
Weinberger et al. (2013)	Knowledge	Tests	Null: the script did not facilitate individual knowledge acquisition.
Wichmann and Rummel (2013)	Artifacts quality	Artifacts	Positive: artifacts quality increased with respect to coherence.
Zimmerman and Land (2022)	Knowledge	Tests	Positive: large gains in science content scores between pre- and post-assessment for the children.

Behavioral outcomes. The second major category of learning outcomes pertains to the behavioral domain (*n* = 20) (as shown in Table 5), including interaction (e.g., discussion, discourse) and engagement (e.g., initiating activities, organizing work, advocating effort). The interaction was the most frequent collaborative learning behavior identified in the selected articles (*n* = 17), exhibiting predominantly positive effects (*n* = 13), along with some studies reporting null effects (*n* = 1) and mixed effects (*n* = 3). While these findings highlight the benefits of collaboration scripts, their impacts on the quality of social interactions remains inconclusive, ranging from positive to non-significant to negative effects (Mahardale and Lee, 2013; Popov et al., 2013; Strauß et al., 2024). Moreover, the negative of scripts in specific domains, such as exchanging resources, have also been observed (Popov et al., 2014). In the domain of engagement, 10 studies investigated the effects of collaboration scripts, consistently reporting positive outcomes (Bouta et al., 2012; Hamalainen, 2011; Miller and Hadwin, 2024; Wang et al., 2017).

**Table 5:** An overview of behavioral learning outcomes in the studies reviewed.

Authors (year)	Type of outcome	Instruments	Findings
Bouta et al. (2012)	Engagement; interaction	User data (log files); tests	Positive: actively engages the students’ interest and leads to richer interaction and results in a higher-level engagement.
Gijlers et al. (2013)	Interaction (discourse)	User data (discourses)	Positive: scripts resulted in higher levels of transactivity in students’ dialogues.
Hamalainen (2011)	Engagement	User data	Positive: all groups engaged in shared collaboration in which students actively provided information and asked questions.
Hämäläinen (2012)	Interaction (shared knowledge construction)	User data (discussions); artifacts (productions)	Positive: a higher amount of shared knowledge construction emerged.
Heimbuch and Bodemer (2018)	Interaction (resolved controversies); engagement (contributions to the discussions)	User data	Positive: more likely to reply to resolved controversies and invested more time for their contributions to the discussions.
Hung et al. (2024)	Interaction	User data	Positive: students are more likely to actively seek assistance from their peers, thereby achieving more frequent interactions.
Mahardale and Lee (2013)	Interaction	User data (messages)	Positive: social groups had greater number of interactions, more elaborated discussions and higher levels of intersubjectivity.
Van der Meij et al. (2020)	Interaction (dialogic acts)	User data (dialogues)	Positive: the script helped raise the quality of the dialogues.
Miller and Hadwin (2024)	Engagement; interaction	User data (response; chat records; log file records)	Positive (engagement): engaged in more transactive negotiation of shared task perceptions Positive (interaction): facilitate group planning interactions.
Noroozi et al. (2012)	Interaction (knowledge transfer)	Artifacts (problem solution plans); user data (messages)	Positive: the transactive memory script facilitated the transfer of domain-specific knowledge; the transactive discussion script facilitated the transfer of domain -specific knowledge.
Noroozi et al. (2013)	Interaction (discussion)	User data (discourses)	Positive: the average scores for formal quality of single arguments were higher for scripted than unscripted learners.
Popov et al. (2013)	Engagement; interaction (discussion)	User data (discussions)	Negative (engagement): the same-culture dyads not using the collaboration script to display a significantly higher frequency of organizing work and initiating activities than the same-culture dyads using the collaboration script. Positive (interaction): the mixed-culture dyads using the collaboration script produced a significantly higher frequency of social interaction. Null (interaction): no significant main effect of script condition on the quality of online discussions.
Popov et al. (2014)	Interaction; engagement	User data (online chats)	Positive (interaction): a higher frequency of Feedback Giving, Sharing Knowledge and Explaining behavior. Negative (interaction): a lower frequency of Social Interaction. Negative (engagement): a lower frequency of Initiating Activities, Organizing Work, Feedback Seeking, Advocating Effort.
Popov et al. (2019)	Interaction; engagement	User data (chat protocols)	Positive (interaction): a higher frequency of Challenging behavior Negative (interaction): a lower frequency of Exchanging resources, Social Interaction. Positive (engagement): a higher frequency of Initiating activities. Negative (engagement): a lower frequency of Organizing work, Sharing knowledge.
Rummel et al. (2012)	Interaction	User data (interaction)	Positive: the scripted dyad shows a higher quality in their collaboration and problem-solving.
Scheuer et al. (2013)	interaction (discussion)	user data (chat protocols)	Positive: an additional proponent/critic role script has a positive influence on the discussion quality.
Strauß et al. (2024)	Interaction	User data (interaction)	Null: did not affect the overall interaction quality.
Wang et al. (2017)	Engagement	User data (discourse)	Positive: increased students’ engagement in meta-cognitive, monitoring and reflection activities.
Wang et al. (2022)	Engagement (behavioral transition)	User data (video analysis)	Positive: the group members exhibited more active and friendly collaborative learning atmosphere.
Wichmann and Rummel (2013)	Engagement (revision behavior); interaction (coordination)	User data	Positive: increased revision behaviors and coordination activities.

Affective outcomes. Out of the 53 studies reviewed, 19 examined the effects of collaboration scripts on the affective domain (see Table 6), including perception (*n* = 4), attitude (*n* = 3), satisfaction (*n* = 4), motivation (*n* = 5), and experience (*n* = 6). While most studies reported positive effects of collaboration scripts on perception (e.g., Chen and Yeh, 2024), attitude (eg., Popov et al., 2019; Rojas et al., 2022), findings regarding satisfaction, motivation and experience were more mixed. Regarding to the satisfaction, although most studies reported positive effects, one study found that lower satisfaction among online participants, who expressed the need for simpler structures and clearer task instructions (Hummel et al., 2015). In terms of motivation, while Peterson and Roseth (2016) found positive effects of collaboration scripts on academic efficacy but negative effects on perceived value, studies by Lin (2020), Van der Meij et al. (2020), and Vogel et al. (2021) reported no significant impact on motivations. These differences may be attributed to variables such as the educational levels of the learners, the duration of the intervention, and the simultaneous existence of both positive and negative effects associated with CSCL scripts (Radkowitsch et al., 2020). With respect to experience, four studies reported positive experiences of learners, while two studies found there were no significant differences between with and without scripted conditions (De Weyer et al., 2015; Rau et al., 2017).

**Table 6:** An overview of affective learning outcomes in the studies reviewed.

Authors (year)	Type of outcome	Instruments	Findings
Chen and Chiu (2023)	Students’ perceptions of activities	Questionnaires	Positive: higher “yes” frequencies
Chen and Yeh (2024)	Perceptions for digital storytelling	Artifacts (reflective essays)	Positive: students’ perceived benefits
Darmawansah et al. (2024)	Attitude (collaboration tendency)	Questionnaires	Positive: significantly enhances the tendency towards collaboration
De Weyer et al. (2015)	Experience for collaboration	Questionnaires	Null:no significant differences were found between the scripted and the non-scripted condition
Heimbuch and Bodemer (2018)	User experience	Questionnaires	Positive: implicit guidance group ≥ explicit guidance group
Hummel et al. (2015)	Satisfaction with collaboration	Questionnaires	Mixed: appreciate to collaborate on these problems in such a playful way, but participants in the online group reported significantly less satisfaction
Lin (2020)	Satisfaction; motivation (self-efficacy; group efficacy; task value)	Questionnaires; artifacts (reports)	Positive (satisfaction): scripted ≥ unscripted; Null (motivation): self-efficacy-did not make any difference; group efficacy-similar levels of group efficacy; task value-not reported
Van der Meij et al. (2020)	Motivation	Questionnaires	Null:same in both conditions.
Miller and Hadwin (2024)	Shared task perceptions	User data (responses)	Positive: more accurate shared task perceptions
Peterson and Roseth (2016)	Motivation	Questionnaires	Mixed: scripting enhanced academic efficacy but diminished value
Popov et al. (2014)	Experience with CSCL	Interview	Positive: more positive CSCL experiences.
Popov et al. (2019)	Attitudes towards online collaboration	Questionnaires	Positive: the change in attitudes towards online collaboration was significantly higher
Rau et al. (2017)	Learning experiences	Questionnaires; interviews	Null: did not affect students’ experiences overall.
Rojas et al. (2022)	Attitude toward collaboration; satisfaction; experience	Questionnaires; interviews	Positive: a positive relationship between the intervention and students’ attitudes, satisfactory and simulating experience
Tsompanoudi et al. (2016)	Experience towards DPP	Questionnaires	Positive: positive; collaborate again in future; the main benefits
Vogel et al. (2016)	Motivation (disposition to use argumentation skills)	Tests	Positive: significant positive effects
Vogel et al. (2021)	Motivation	Questionnaires	Null: non-significant effects
Wang et al. (2023)	Group-process satisfaction	Questionnaires	Positive: students provided external scripts had higher GS
Wichmann & Rummel (2013)	Perception of wiki-based writing setting	Questionnaires	Positive: students perceived the main components, the wiki-page and wiki-editing as useful

Skills-based outcomes. Eighteen studies (see Table 7) investigated the skills developed by learners through CSCL supported by collaboration scripts, encompassing higher-order thinking skills (*n* = 7), higher-order thinking skills (*n* = 7), social and collaborative skills (*n* = 3), and domain-specific skills (*n* = 3). All of the included studies identified the positive impacts of collaboration scripts in higher-order thinking skills, such as critical thinking and problem-solving proficiency, demonstrating that scripts helped learners engage in deeper analysis and reasoning during collaborative tasks. Similarly, domain-specific skills, referring to knowledge and competencies of learners in specific subjects, such as mathematical literacy (Chen and Chiu, 2023), writing skills (Chen and Yeh, 2024), and science reading literacy, were consistently enhanced. In contrast, the impact of collaboration scripts on regulation-related skills (self-regulation and social regulation) as well as social and collaborative skills was mixed, with some studies reporting significant improvements (Hung et al., 2024; Rojas et al., 2022) while others found no effect (Chen and Chiu, 2016; Lin, 2020).

**Table 7:** An overview of skills-based learning outcomes in the studies reviewed.

Authors (year)	Type of outcome	Instruments	Findings
Chen and Chiu (2016)	Domain-specific skills (mathematics literacy); regulation-related skills (metacognitive self-regulation)	Tests; questionnaires	Positive (domain-specific skills): better learning outcomes in higher level instead of lower level of questions Mixed (regulation-related skills): better metacognitive self-regulation in the controlling aspect but no statistically significant differences in the planning aspect
Chen and Chiu (2023)	Social and collaborative skills (collaborative skills)	Questionnaires	Positive: students learning with the scripts under intergroup competition demonstrated better collaborative skills than their counterparts who did not experience intergroup competition.
Chen and Yeh (2024)	Domain-specific skills (writing skills); higher-order thinking skills (graphic creativity)	Tests	Positive: higher writing skills; higher imaginary creativity scores
Darmawansah et al. (2024)	Higher-order thinking (argument skills, critical thinking awareness)	Tests; questionnaires	Positive: a clear progression in argumentation skills; significantly enhances EFL students’ critical thinking awareness
Hung et al. (2024)	Social and collaborative skills (communication skills)	Questionnaires	Positive: significant improvement in the experimental groups and communication skills
Kielstra et al. (2022)	Regulation-related skills (metacognitive regulation)	User data (conversations); tests	Positive: elicited metacognitive regulation in the groups; students did improve on individual metacognitive regulation
Lee (2015)	Domain-specific skills (science reading literacy)	Tests mid-term scores	Positive: C-QRAC group performed significantly better than the control students
Lin (2020)	Social and collaborative skills (social ability)	Questionnaires	Null: no significant main effects
Mahardale and Lee (2013)	Higher-order thinking skills (problem-solving proficiency)	Artifacts (solutions)	Positive: social groups are better than in epistemic groups and enable students’ group problem-solving performance
Mende et al. (2017)	Higher-order thinking skills (deep text comprehension)	Tests; user data (discussions)	Positive: low prior knowledge learners benefited from the high guidance scripts, whereas high prior knowledge learners profited from the low guidance scripts
Peterson and Roseth (2016)	Regulation-related skills (socio-cognitive conflict regulation)	Questionnaires	Positive: greater epistemic conflict regulation
Ramirez and Monterola (2022)	Higher-order thinking skills (logical thinking)	Tests	Positive: CSCL with scripting significantly influenced the development of logical thinking
Raes et al. (2016)	Regulation-related skills (metacognitive awareness, socially shared metacognitive regulation)	Questionnaires	Mixed: positive – the overall implementation improved students’ meta cognitive awareness; no significant improvement in socially shared regulation was found that could be attributed to the classroom script intervention
Rojas et al. (2022)	Regulation-related skills (regulation skills)	Interviews	Positive: the proposed collaborative game develops regulation skills
Rummel et al. (2012)	Higher-order thinking skills (problem-solving)	User data	Positive: the scripted dyad shows a higher quality in their problem-solving than the unscripted dyad.
Stegmann et al. (2012)	Higher-order thinking skills (argumentation)	User data (discussions)	Positive: the quality of argumentation can be fostered during collaboration by means of a computer-supported collaboration script.
Vogel et al. (2022)	Higher-order thinking skills (argumentation skills)	Tests	Positive: significant effect of the script condition on students’ acquisition of domain-specific MAS
Wang et al. (2017)	Regulation-related skills (regulation skills)	User data (discourse)	Positive: had a positive effect on students’ acquisition of regulation skills

### 3.3 Measurements of learning outcomes

**Figure 7:**
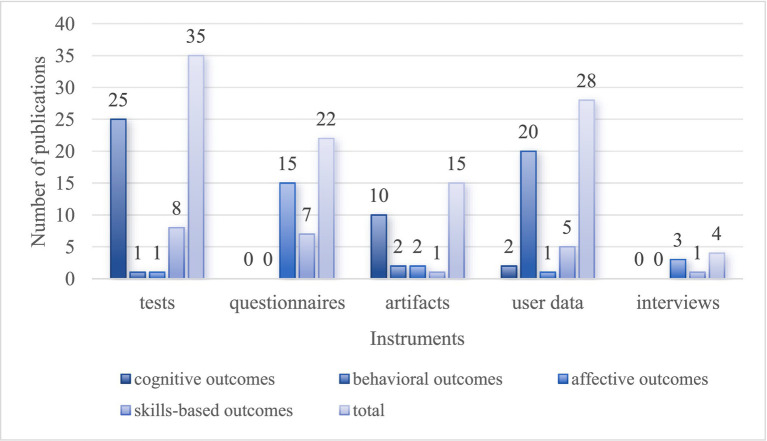
Distribution of articles on collaboration scripts in CSCL by instruments (2011–2024).

Mixed methods with both quantitative and qualitative evaluation approaches were used to measure learning outcomes in the cognition, behavior, affection and skills. As shown in Figure 7, five instruments have been identified for measuring four domains of learning outcomes, which were tests, questionnaires, artifacts, user data, interviews. The tests were the most frequently applied (*n* = 35), while interviews were the least prevalent measurement (*n* = 4).

Tests, which comprised a range of established standardized tests, school examinations and questions developed by teachers or experts, were applied in all four categories of learning outcomes. Especially, the cognitive domain (*n* = 25) and the skills-based domain (*n* = 8) were the most predominantly employed. A series of studies have been conducted to compare the acquisition and comprehension of specific domain knowledge by means of pre-tests and post-tests to ascertain the effectiveness of collaboration scripts. For example, Chen and Yeh (2024) measured learners’ vocabulary acquisition by pre- and post-vocabulary acquisition tests.

Twenty-eight publications used user data as one of the tools for assessing learning outcomes, particularly in the behavioral domain (*n* = 20). The user data refers to the information generated and recorded during the collaborative process, such as messages, interactions, dialogical acts, and responses, which were analyzed by quantitative and qualitative analyses. Firstly, it can be quantitatively recorded and analyzed through educational technology tools to assess and explain learners’ performance, engagement, and interactions. For example, Mahardale and Lee (2013) used the total messages of triads to evaluate the patterns of interactions. Secondly, the content of chats, discussions and discourses during collaboration can also be qualitatively analyzed using specific coding schemes to address research questions and provide deeper insights into the nature of the collaboration. For example, the coding scheme developed by Curtis and Lawson (2001) was the most frequently employed to categorize collaborative behaviors in this review (Popov et al., 2013; Popov et al., 2019). To illustrate the transactivity, Gijlers et al. (2013) classified utterances into five distinct categories, drawing upon frameworks developed by Weinberger and Fischer (2006).

We observed that the questionnaires have been widely utilized within two domains: affective outcomes (*n* = 15) and skills-based outcomes (*n* = 7). The questionnaires used in selected articles were generally adopted by mature scales with Likert format for quantitative measurement of learning outcomes. For example, Popov et al. (2019) used a 17–item questionnaire adopted by Thompson et al. (2006) to measure the students’ attitudes towards online collaboration in a 5-point Likert format. Meanwhile, open-ended questionnaires were also used for qualitative measurement of learning outcomes, and students’ open-ended responses would be coded by at least two independent researchers using the coding scheme (e.g., Chen and Chiu, 2023; Rau et al., 2017).

The artifacts were a lot of products, such as reports, essays, assignments, solutions and presentations, which were completed during the process of collaboration and which demonstrated the changes in academic performance. A majority of the articles (n = 10) focused on examining learning outcomes within the cognitive domain (Chen and Yeh, 2024; Miller and Hadwin, 2024), while one publication explored skills-based outcomes through the analysis of artifacts.

Interview were mainly applied in assessing affective outcomes (*n* = 3) and skills-based outcomes (*n* = 1). These studies provided insights into the experiences and attitudes of learners regarding scripts and collaboration learners (Rau et al., 2017; Rojas et al., 2022).

### 3.4 Factors influencing the impacts of collaboration scripts on learning outcomes

**Figure 8:**
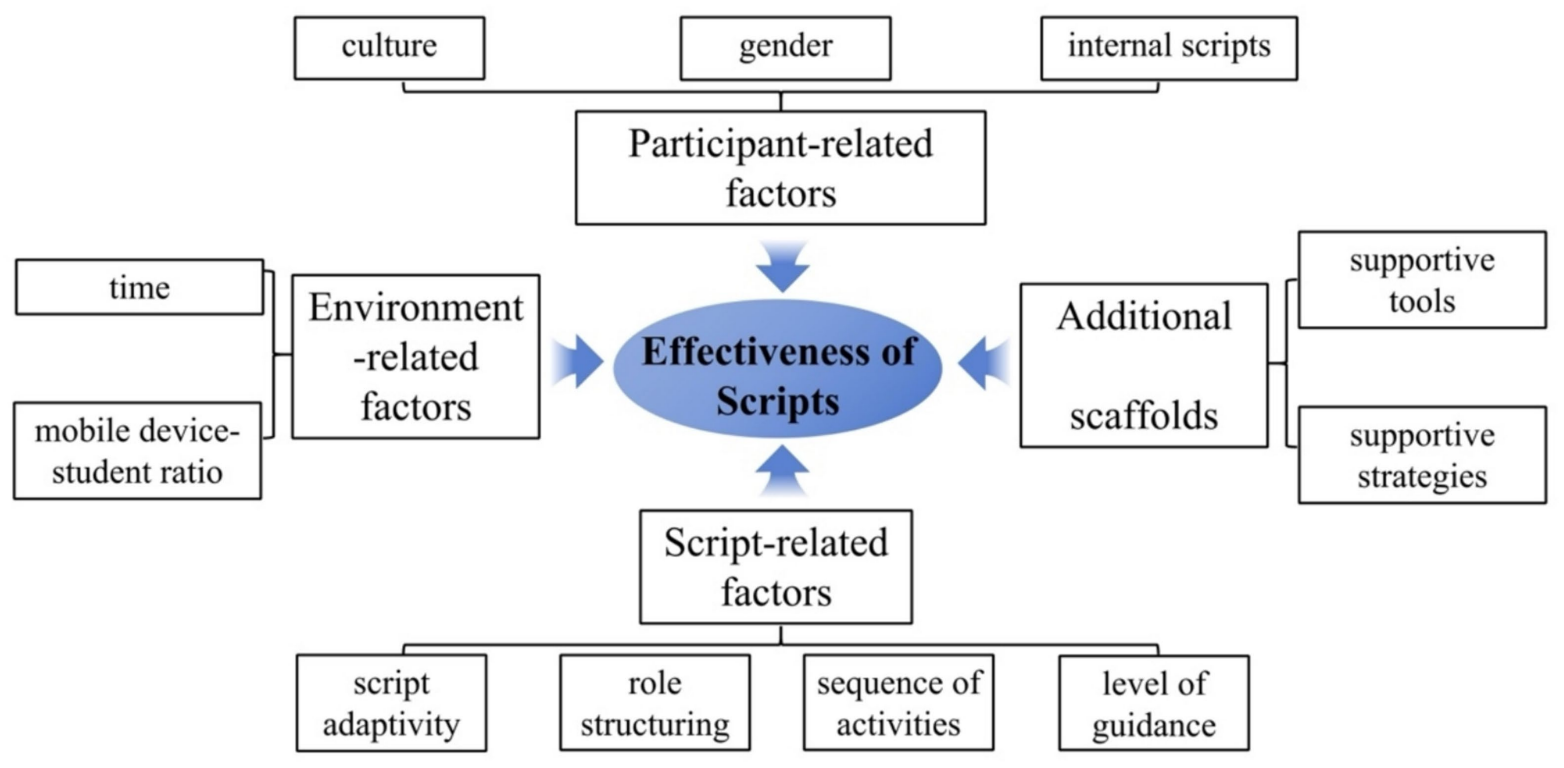
The model of influencing factors of collaboration scripts.

To address RQ4, the factors influencing the impact of collaboration scripts identified across the included studies were synthesized through inductive content analysis. This synthesis resulted in a four-category model (see Figure 8), comprising environment-related factors, participant-related factors, script-related factors, and additional scaffolds, along with 11 subcategories.

#### 3.4.1 Environment-related factors

Environment-related factors include time and the mobile device–student ratio. Time was identified as a factor shaping the impact of scripts on knowledge acquisition and cognitive load. For example, although students with external script support initially demonstrated greater knowledge acquisition than those without such support, this advantage decreased over time (Wang et al., 2023). As learners become more familiar with collaborative processes, reliance on externally provided prompts may diminish. The mobile device–student ratio also shaped the reported impact of external scripts on group interaction. In the 1:m condition, external scripts were associated with more positive behavioral engagement and enhanced information exchange (Wang et al., 2022). Compared with the 1:1 condition, the 1:m setting appeared to lower the technological adaptation threshold by encouraging students to collaborate around a common focus (a shared device), thereby fostering more efficient interaction.

#### 3.4.2 Participant-related factors

Participant-related factors include culture, gender, and learners’ internal scripts (e.g., prior knowledge and self-regulation skills).

Cultural background was reported to shape how students engage with collaboration scripts. Weinberger et al. (2013) observed that Finnish students more frequently integrated partners’ arguments, whereas German students adopted a more conflict-oriented style, which was linked to broader cultural discourse norms. Similarly, Popov et al. (2013) found that same-culture dyads outperformed mixed-culture dyads under scripted conditions. Gender was also identified as a potential moderating factor. Lee (2015) reported that male students demonstrated greater gains than female students in science reading literacy tasks when working with scripted support. Learners’ internal scripts, including prior knowledge and self-regulation skills, were frequently reported as moderators of script impact. For example, prior knowledge influenced how learners benefited from different levels of guidance (Mende et al., 2017). Learners with lower prior knowledge tended to benefit more from highly guided scripts, whereas those with higher prior knowledge showed greater gains under minimally guided conditions. Similarly, learners with stronger self-regulation skills were more likely to benefit from adaptable CSCL scripts (Vogel et al., 2022).

#### 3.4.3 Script-related factors

Script-related factors include script adaptivity, role structuring, sequence of activities, and level of guidance.

Studies examining fade-out scripts, which gradually reduce instructional support during the learning process, reported no significant differences in domain learning, possibly because the underlying Toulmin model was readily understood and internalized by students (Bouyias and Demetriadis, 2012). Adaptable scripts, which allow learners to regulate the level of guidance received, were associated with improved outcomes in areas such as mathematical argumentation, though these impacts were moderated by learners’ self-regulation abilities (Vogel et al., 2022; Wang et al., 2017). Self-adaptive scripts, adjusted automatically based on learner performance, were reported to support understanding of complex concepts (Rau et al., 2017).

Role structuring refers to the extent to which collaboration scripts specify clear and explicit role assignments among group members. Studies generally reported more positive outcomes when roles were clearly specified. For example, explicit role assignments (e.g., Adviser, Organizer, Innovator) were associated with enhanced interaction quality and problem-solving performance (Mahardale and Lee, 2013). Similarly, the Peer-Critique Script with complementary roles (Proponent and Constructive Critic) improved the quality of online discussions (Scheuer et al., 2013). However, the reported effects of roles were moderated by task difficulty: for low-difficulty tasks, the I-to-C mode without role assignment outperformed the R-to-C mode in reducing errors, whereas for high-difficulty tasks, the structured role assignment in R-to-C led to fewer errors than I-to-C (Dlab et al., 2020).

The sequence of activities also shaped collaborative processes. For example, the “Discuss, Deliberate, Revise” (DDR) script was associated with higher knowledge test scores and fewer errors compared with the “Be Bold, Revert, Discuss” (BRD) script, potentially because it guided collaboration toward deeper knowledge building (Heimbuch et al., 2018).

The level of guidance further influenced reported impacts. Higher guidance did not consistently lead to stronger outcomes, as its impact depended on contextual factors, such as learners’ prior knowledge. For instance, Mende et al. (2017) reported that learners with low prior knowledge showed greater gains under high-guidance scripts, whereas learners with high prior knowledge demonstrated stronger outcomes under low-guidance scripts. Vogel et al. (2021) found that a mid-level scripting approach was associated with optimal argumentation knowledge acquisition, outperforming both the play-level and scriptlet-level scripts.

#### 3.4.4 Additional scaffolds

Additional scaffolds include supportive tools and structured strategies integrated with collaboration scripts.

Supportive tools (e.g., argument builders, representational tools, group awareness tools, and planning tools), defined as software or technological features embedded in collaborative learning environments, were frequently reported to support collaborative processes and learning outcomes across cognitive and affective domains. These tools served diverse functions within scripted collaboration contexts. For example, the argument builder was described as a structured presentation tool that enabled students to reflect on and organize their ideas more systematically, and was associated with improvements in interaction quality and learning outcomes (Cáceres et al., 2018). Representational tools, including causal and simulation tools, were reported to support collaborative quality and task performance in complex learning contexts by facilitating qualitative and quantitative reasoning when integrated with collaboration scripts (Slof et al., 2012). Group awareness tools provided learners with information to facilitate the enactment and coordination of collaboration scripts, and were associated with enhanced cognitive, affective, and social outcomes (Rojas et al., 2022). Planning support tools were described as guiding learners in developing shared task representations during the initial planning phase, thereby supporting collaborative regulation and contributing to improved learning processes (Miller and Hadwin, 2024). More recently, generative AI tools have been explored as additional scaffolds within scripted collaboration. For instance, integrating ChatGPT into collaborative projects was reported to support students’ argumentative skills and the quality of their argumentation, particularly through providing informational support and individualized guidance (Darmawansah et al., 2024).

Supportive strategies refer to structured methods and approaches that guide collaborative processes, including content schemata, summarization, peer monitoring, and intergroup competition mechanisms. Several studies described interactive patterns between collaboration scripts and these strategies. For example, Peterson and Roseth (2016) reported that summarization, when combined with scripts in CSCL, was associated with improvements in motivation, peer relations, and academic achievement, as it provided clearer task-related information that supported the enactment of prescribed roles and procedures during collaboration. Similarly, content schemata integrated with collaboration scripts were reported to support improved task solutions by deepening argumentative processes and facilitating the internalization of strategies (Kopp and Mandl, 2011). In scripted argumentation contexts, peer monitoring was associated with enhanced domain-specific learning, as it reinforced script guidance and encouraged more active participation in knowledge construction (Bouyias and Demetriadis, 2012). Likewise, the integration of collaboration scripts with inter-group competition mechanisms was reported to support teamwork and collaboration skills, particularly by addressing limitations observed when relying solely on scripts, such as overly formalized execution (Chen and Chiu, 2023).

## 4 Discussion

### 4.1 Characteristics of selected studies

The fluctuating trajectory of collaboration script research over the past 14 years reflects evolving theoretical debates regarding the role of external structuring in collaborative learning. The decline observed after 2013 may correspond to growing concerns that highly structured scripts could constrain learners’ autonomy, thereby potentially interfering with motivational and self-regulatory processes (Kaliisa et al., 2025; Radkowitsch et al., 2020). The renewed interest after 2020, particularly in adaptive scripts and integrations with Generative Artificial Intelligence (GenAI), indicates a theoretical shift toward dynamic regulation and context-sensitive scaffolding to mitigate the limitations of over-scripting rather than static procedural guidance (Darmawansah et al., 2024; Vogel et al., 2022). In this sense, the evolution of script research may reflect increasing theoretical attention to models that integrate self-regulation, co-regulation, and socially shared regulation, often supported by adaptive technologies (Panadero, 2017).

The dominance of European contexts, alongside the gradual increase of studies from Asia and the Americas, suggests that collaboration script design may be culturally embedded rather than universally neutral. Because collaboration scripts structure discourse norms, role expectations, and regulatory moves, they implicitly encode culturally grounded assumptions about interaction, authority, and epistemic participation. The limited representation of Global South contexts therefore restricts the generalizability of the script-guided collaboration across diverse sociocultural settings.

With regard to research design, the strong prevalence of experimental and quasi-experimental studies reflects an emphasis on isolating causal relationships between external scripting and learning outcomes. However, most studies were conducted in controlled environments with relatively small groups, which may constrain the applicability of findings.

The predominance of relatively small samples (*n* < 200, with groups typically fewer than six members) reflects the intensive and fine-grained nature of script-based CSCL research. While such designs enable close analysis of regulatory processes, they may limit statistical power and the robustness of effect estimations. Notably, nearly 28% of the reviewed studies included fewer than 50 participants, raising concerns about the stability and generalizability of reported findings (Bouta et al., 2012; Hämäläinen, 2012; Hummel et al., 2015). This pattern suggests that current evidence on script effectiveness may be shaped not only by theoretical assumptions but also by practical design constraints inherent in small-scale experimental research.

The concentration of collaboration script research in higher education further reflects an implicit assumption regarding learners’ readiness for structured collaborative regulation. University students are typically more experienced in ICT use and academic discourse practices, which may make them more responsive to externally structured scripting interventions. However, this focus raises important developmental considerations. The effectiveness of scripts may depend on learners’ prior collaborative competencies, digital literacy, and meta-cognitive maturity. The limited representation of K–12 contexts therefore constrain understandings of how age-related and educational-level differences shape engagement with scripted collaboration.

### 4.2 Learning outcomes affected by collaboration scripts

The reviewed studies reveal that collaboration scripts are associated with learning outcomes across cognitive, behavioral, affective, and skills-based domains.

The predominance of cognitive outcomes reflects a traditional emphasis on knowledge acquisition and conceptual change. However, the mixed patterns reported across studies highlight that cognitive gains are not solely determined by the presence of structural guidance. Instead, they appear contingent upon how scripts interact with learners’ prior knowledge, task complexity, technological affordances, and opportunities for epistemic engagement. These findings suggest that scripts function as regulatory tools whose influence depends on the alignment between external structure and learners’ internal cognitive resources. Interpreted through a cognitive load perspective, rigid or overly detailed scripts may impose extraneous load that competes with generative processing (Darmawansah et al., 2024; Vogel et al., 2022), whereas optimally calibrated scripts may reduce unnecessary coordination demands and free resources for deeper conceptual integration. Therefore, scripts do not uniformly enhance cognition, and their effectiveness depends on how well they align with cognitive demands.

In the behavioral domain, scripts consistently shape interaction frequency and engagement patterns, indicating their role in structuring observable regulatory moves. However, improvements in interaction quality are less consistent. This divergence highlights a psychologically meaningful distinction between surface-level coordination and deep co-construction of knowledge. In short-term interventions, learners may comply procedurally with scripted guidance without internalizing the underlying regulatory principles, resulting in behavioral alignment without substantive transformation (Strauß et al., 2024). Moreover, scripts cannot be treated as culturally neutral tools, and their impact is mediated by the sociocultural conditions that shape how learners interpret, negotiate, and enact structured interaction (Popov et al., 2013). Accordingly, scripts may organize interactional form, but they do not necessarily enhance epistemic depth unless sufficient time and cultural alignment support the internalization of regulatory processes.

Affective outcomes further illuminate the motivational dynamics underlying scripted collaboration. Reports of diminished task value or reduced satisfaction when scripts are perceived as controlling suggest that external regulation can conflict with learners’ autonomy needs (Hummel et al., 2015; Peterson and Roseth, 2016). This tension reflects the delicate balance between guidance and self-determination. Excessive structuring may undermine perceived agency, whereas insufficient guidance may fail to support competence. From the perspective of Self-Determination Theory, autonomy, competence, and relatedness are fundamental psychological needs underlying sustained motivation (Ryan and Deci, 2017). Collaboration scripts may therefore operate as either autonomy-supportive structures that clarify competence and promote balanced participation, or as controlling constraints that undermine intrinsic engagement. The mixed affective evidence suggests that the motivational impact of scripting depends not simply on the presence of structure, but on how that structure is experienced and internalized by learners.

The growing emphasis on higher-order thinking skills reflects a holistic view of learners as active constructors or problem-solvers of knowledge. However, the mixed findings regarding regulation-related and social skills suggest that collaboration scripts do not consistently support deeper regulatory and collaborative competencies. For instance, non-significant improvements on planning may result from insufficient sustained guidance throughout the regulatory process (Chen and Chiu, 2016), while the limited impact on socially shared regulation and social ability may stem from narrowly defined script roles, added cognitive load, or inadequate contextual support (Lin et al., 2020; Raes et al., 2016).

### 4.3 Measurements of learning outcomes

Five primary evaluation methods—tests, questionnaires, user data, artefacts, and interviews—were identified, reflecting the multidimensional nature of learning outcomes in script-based CSCL. The prevalent use of pre–post designs with reported reliability indices (Heimbuch et al., 2018; Peterson and Roseth, 2016; Van der Meij et al., 2020) indicates considerable methodological rigor. However, the dominance of single-method assessments limits our ability to capture the psychological processes underlying observed performance gains.

From a psychological perspective, the reliance on single outcome measures limits our understanding of how collaboration scripts influence underlying learning mechanisms. Although scripts are theorized to shape not only observable performance but also internal regulatory processes, motivation, and socially shared cognition, studies that focus exclusively on achievement scores or self-report measures risk conflating behavioral compliance with deeper cognitive transformation. Such approaches often overlook process-level dynamics—such as regulatory transitions, epistemic moves, and affective fluctuations—that unfold during interaction. The limited triangulation across behavioral traces, discourse analysis, and subjective experiences therefore constrains our ability to reconstruct how scripts reshape collaborative regulation over time and may partly explain inconsistencies in reported effects across studies.

### 4.4 Influencing factors of collaboration scripts

The impacts of collaboration scripts do not depend solely on their structural design, but on a constellation of factors that shape how scripted guidance is interpreted and enacted. The reviewed evidence suggests four interrelated categories of influencing factors: environment-related factors, participant-related factors, script-related factors, and additional scaffolds. Rather than functioning independently, these factors interact to determine whether external scripting translates into sustained regulatory engagement. This perspective shifts attention from asking whether scripts work to examining the conditions under which they foster meaningful collaborative regulation.

Environment-related factors establish the macro-level conditions for collaborative regulation. Although some studies suggest that the effects of collaboration scripts may diminish over time (Wang et al., 2023), time has rarely been examined as a psychological variable influencing internalization and sustainability of regulation. Similarly, device configuration influences interaction dynamics (Wang et al., 2022). However, the mechanisms through which technological arrangements affect regulatory alignment remain under explored. Therefore, future research should re-conceptualize time and technological affordances as developmental and structural conditions that interact with learners’ regulatory capacities and task demands to shape collaborative cognition.

Participant-related factors—including culture, gender, prior knowledge, and self-regulation skills—underscore the importance of aligning external scripts with learners’ internal scripts. Learners enter collaboration with prior cognitive and motivational regulatory patterns that shape how scripted guidance is interpreted and enacted (Fischer et al., 2013; Mende et al., 2017). These internal scripts influence whether externally structured prompts are transformed into meaningful co-regulation or enacted mechanically. When external scripts are congruent with learners’ internal regulatory resources, they can amplify engagement and deepen knowledge construction. When misaligned, however, scripts may exceed learners’ regulatory capacities—leading to passive participation—or constrain advanced learners by limiting autonomy and epistemic challenge. Cultural norms further shape this alignment process by influencing expectations for argumentation and participation (Popov et al., 2013).

Script-related factors—including adaptivity, role structuring, activity sequencing, and guidance level—operate as mechanisms for distributing and gradually transferring regulatory control within collaborative settings. Their effectiveness depends on learners’ evolving self- and socially shared regulation: without sufficient regulatory capacity, even adaptive structures may result in procedural compliance rather than meaningful co-regulation (Cai et al., 2025; Vogel et al., 2022). In this sense, script design exerts its influence indirectly, through alignment with learners’ internal regulatory resources, rather than as a uniform driver of learning outcomes.

Based on participant- and script-related factors, future research should investigate how these elements jointly shape the dynamic alignment between external guidance and learners’ internal regulation. Longitudinal, process-oriented designs could trace how self- and socially shared regulation co-develop with adaptive script mechanisms over time. Specially, AI-driven adaptive scripts offer a promising way to operationalize this alignment: intelligent systems could monitor learners’ regulatory processes in real time and adjust prompts, task difficulty, and feedback to optimize engagement, autonomy, and co-regulation.

Additional scaffolds—including supportive tools and instructional strategies—play a complementary role in enhancing collaboration scripts. Tools such as argument builders, representational systems, group awareness dashboards, planning supports, and generative AI extend and enrich scripts by structuring reasoning, enhancing transparency, and supporting individualized planning. Supportive strategies (e.g., content schemata, summarization, intergroup competition, peer monitoring) further regulate task engagement, motivation, and participation equity. These scaffolds may function as mediators that strengthen regulatory alignment by compensating for learners’ internal limitations or contextual constraints. However, current evidence does not clarify whether effectiveness depends on the quantity of scaffolds, their type, or their congruence with learners’ internal scripts. Future empirical work should therefore examine boundary conditions and interaction effects, investigating how combinations of scaffolds influence regulatory processes rather than solely performance outcomes.

## 5 Implications

### 5.1 Theoretical implications

The findings of this review contribute to psychological understanding of computer-supported collaborative learning in several ways. First, the synthesis conceptualizes collaboration scripts as external regulatory structures that redistribute control within collaborative activity systems. Rather than functioning merely as procedural guidance, scripts shape learners’ cognitive, behavioral, and affective processes. The mixed findings across domains indicate that their effectiveness depends on the degree of fit between externally provided regulation and learners’ internal regulatory capacities, supporting a dynamic view of collaborative regulation. Second, the affective variability observed across studies can be interpreted through Self-Determination Theory. Scripts may operate as autonomy-supportive structures that clarify expectations and enhance perceived competence, or as controlling constraints that undermine agency. These differences suggest that motivational outcomes hinge on how learners interpret and internalize externally imposed structure within collaborative processes. Third, the identified influencing factors further indicate that scripting impacts are context-sensitive. Cultural norms, prior knowledge, motivational orientations, and technological affordances shape how external structure is enacted and experienced. Consequently, the impact of collaboration scripts emerges from interactions among learners, tasks, and environments rather than from design features alone. In a word, collaboration scripts can be understood as regulation-distribution mechanisms whose cognitive and motivational consequences arise from the interplay between structural guidance and learners’ psychological and contextual conditions.

### 5.2 Practical implications

The findings of this review offer several guidance for educators and instructional designers. First, script design should prioritize alignment between structural guidance and learners’ developmental and regulatory capacities. Rather than uniformly increasing guidance, designers should calibrate the level of structure to avoid over-regulation that may constrain autonomy or under-regulation that may leave learners without sufficient support. Second, educators should consider allocating sufficient collaboration time to allow learners to internalize externally provided roles and prompts. Short-term implementation may result in surface-level enactment without deeper regulatory transformation. Third, adaptive scripting mechanisms should be accompanied by supports that enhance learners’ meta-cognitive awareness and reflection, as adaptivity alone may not ensure effective engagement. Integrating planning tools, awareness dashboards, or reflective prompts may facilitate the gradual transfer of regulatory responsibility. Fourth, evaluation practices should extend beyond outcome performance. Educators and researchers are encouraged to combine achievement measures with process-tracing methods—such as discourse coding, log-file analyses, and temporal sequence analysis—to capture how regulatory transitions unfold during collaboration.

## 6 Limitations and future directions

This review is not without limitations. Firstly, as with many systematic reviews, publication bias may be present. Most included studies emphasize positive impacts on cognitive performance, while fewer report non-significant findings across domains. It is possible that studies with null or negative results remain unpublished. Moreover, this review was restricted to peer-reviewed journal articles written in English, thereby excluding other sources such as conference papers, book chapters, and doctoral dissertations. Future research may address this limitation by conducting broader scoping reviews that include diverse publication types and linguistic contexts.

Secondly, the model of influencing factors was constructed inductively. Although systematic coding procedures were applied, some degree of interpretative subjectivity is unavoidable. In addition, the relatively limited literature base constrained the hierarchical refinement of the model. Future studies should empirically test and extend this framework, particularly by examining how identified factors interact dynamically within collaborative processes and by grounding such models more explicitly in psychological theories of regulation, motivation, and cognitive development.

Finally, the methodological scope of this review establishes boundaries. The synthesis was primarily descriptive and qualitative rather than quantitative and does not provide statistical estimations of effects magnitude across domains. Evidence remains comparatively scarce in affective and skills-based areas. Future research should therefore employ diverse methodological approaches, including longitudinal designs, process-oriented analyses, and meta-analytical techniques, to clarify domain-specific patterns and to further unpack the psychological mechanisms underlying scripted collaboration.

## 7 Conclusion

This review synthesized evidence from 53 empirical studies on collaboration scripts in CSCL published between 2011 and 2024, offering a comprehensive examination of research characteristics, learning outcomes, measurement approaches, and influencing factors. Beyond mapping empirical patterns, the findings provide theoretical insight into how externally structured collaboration shapes learning processes.

Across domains, collaboration scripts were associated with cognitive, behavioral, affective, and skills-based outcomes. However, reported effects were neither uniform nor universally positive. While scripts consistently structured interaction frequency and engagement, improvements in epistemic depth, motivation, and regulatory skills were more variable. These inconsistencies suggest that the effectiveness of scripting cannot be explained by structural guidance alone.

A central contribution of this review lies in conceptualizing collaboration scripts as regulation-distribution mechanisms. Rather than functioning as static instructional tools, scripts redistribute regulatory control within collaborative systems. Their impact depends on the dynamic alignment between externally provided structure and learners’ internal regulatory capacities, such as prior knowledge and self-regulation skills. When external and internal scripts are congruent, structured guidance can facilitate co-regulation and deeper knowledge construction. When misaligned, scripting may result in superficial compliance and cognitive overload.

The inductively derived model of influencing factors further underscores the contextual and interactive nature of scripting effects. Environmental conditions, participant characteristics, script features, and additional scaffolds jointly shape whether external regulation is internalized, negotiated, or resisted. In this sense, the impact of collaboration scripts emerges from the interplay between structural design and psychological processes rather than from design features alone.

Overall, this review advances psychological understanding of scripted collaboration by shifting the focus from whether scripts work to how and under what conditions externally distributed regulation becomes meaningfully integrated into learners’ self- and socially shared regulatory systems. In particular, the findings highlight that the impact of collaboration scripts depends on the alignment among external regulatory structures, learners’ internal regulatory capacities, and contextual conditions.
